# Attainment of smiling and walking in infancy associates with developmental delays at school entry in moderately-late preterm children: a community-based cohort study

**DOI:** 10.1186/s12887-021-02548-9

**Published:** 2021-02-17

**Authors:** Nienke H. van Dokkum, Sijmen A. Reijneveld, Arend F. Bos, Marlou L. A. de Kroon

**Affiliations:** 1 Department of Pediatrics, Division of Neonatology, Beatrix Children’s Hospital, University Medical Center Groningen, University of Groningen, Hanzeplein 1, 9713 GZ Groningen, The Netherlands; 2 Department of Health Sciences, University Medical Center Groningen, University of Groningen, Hanzeplein 1, 9713 GZ Groningen, The Netherlands

**Keywords:** Developmental delay, Developmental milestones, Moderately-late prematurity

## Abstract

**Background:**

Moderately-late preterm (MLP) children (gestational age [GA] 32–36 weeks) are followed-up within community services, which often use developmental milestones as indicators of delay. We aimed to examine associations of parental report of smiling-age and walking-age with developmental delay upon school entry for MLP and full-term children.

**Methods:**

This study regards a community-based cohort study, including 1241 children. Parent-reported smiling-age (*n* = 514) and walking-age (*n* = 1210) were recorded in preventive child healthcare. To determine developmental delay at school entry (at age 4) we used the Ages and Stages Questionnaire (ASQ) total and domain scores. We assessed the association of smiling-age and walking-age with dichotomized ASQ-scores, using logistic regression analyses.

**Results:**

For MLP children, each week later corrected smiling-age was associated with a relative increased likelihood of delays of 31, 43, 36 and 35% in the personal-social, problem-solving, gross motor and general developmental functioning, respectively. Each month later corrected walking-age was associated with a relative increased likelihood of delays of 10, 15 and 13% in the personal-social, gross motor and general developmental functioning, respectively. All corrected smiling-ages and walking-ages were within normal full-term ranges. For full-term children, we only found that later walking-age was associated with delays in the personal-social and gross motor domains.

**Conclusions:**

Smiling-age and walking-age are associated with developmental delay in several domains for MLP and full-term children. Professionals could use these milestones to identify children that may benefit from closer monitoring of their development.

**Trial registration:**

**Clinical Trial Registry name and registration number**: controlled-trials.com, ISRCTN80622320.

## Introduction

Approximately 10% of all children are born preterm, of which a large majority (80–85%) are moderately-late preterm (MLP, gestational age [GA] 32–36 weeks) [1]. MLP birth may have consequences for motor and cognitive development [2–4]. Close monitoring may lead to early identification of these consequences, thereby expediting timely interventions and possibly preventing sequelae into adulthood [5]. Because MLP children are often followed-up within community-based pediatric care, one commonly implemented method regards monitoring of developmental milestone attainment [5].

For full-term children, attainment ages of various developmental milestones have been firmly established, [6] and studies report associations of attainment ages with educational level and intelligence quotient (IQ) in later life [7, 8]. One important early developmental milestone is the attainment age of “smiling in response” (hereafter: ‘smiling-age’). For full-term children smiling-age has been reported to be associated with cognitive development in later life [9]. Another important milestone is the attainment age of “walking independently” (hereafter: ‘walking-age’) [10–13]. Again for full-term children, an association has been reported between walking-age and IQ in toddlerhood and at school-age [14, 15]. These two milestones are precisely recorded in Dutch preventive child healthcare (PCH) in weeks and months respectively. MLP children have been shown to have a higher frequency of delayed attainment of most milestones using chronological age assessments [16]. This delay is attributed to a shorter post-conception age at birth as well as altered brain development outside the womb due to perinatal problems, [17, 18] and possibly an earlier influence of the environment [17].

To the best of our knowledge, for MLP children, attainment of these two important milestones and their value as indicators of developmental delay upon school entry is unknown. Nonetheless, particularly for MLP children, understanding the associations between early developmental milestones and later developmental delay may lead to closer monitoring, enabling earlier identification of developmental delays and offering timely targeted preventive interventions [5]. In the Dutch setting, the majority of MLP children are followed-up in Preventive Child Healthcare Centers (PCHCs), which is an ultimate setting to study developmental milestone attainment for this GA group. In this study our aim was, therefore, to examine the associations of attainment ages of smiling and walking with developmental delay in multiple domains in MLP and full-term children upon school entry (age 4 years).

## Patients and methods

### Setting and population

For our study we used data from the Longitudinal Preterm Outcome Project (LOLLIPOP). LOLLIPOP is a community-based cohort including MLP children, as well as early preterm and full-term children. Children were enrolled in this study at age 4. Thirteen PCHCs participated in the study, from which 45,446 children (comprising almost 25% of all Dutch 4-year-olds) were screened for eligibility. All children with a GA < 36 weeks were included. After every second preterm child, the next full-term child from the same birth year was selected as a control. The children were included via 13 PCHCs covering 25% of the Dutch population with some overrepresentation of the Eastern half of the Netherlands. All five NICUs operating in this catchment area were included as well. One of the initial aims of the LOLLIPOP study was to construct growth charts for every gestational age week, with a particular focus on MLP children. Therefore, MLP and full-term controls were sampled in a 2:1 ratio.

Upon inclusion, children with major congenital malformations and syndromes were considered ineligible. Of the 2517 eligible children, 1983 participated in LOLLIPOP (response 79%), of which 927 were MLP, 544 were full-term children, and the remaining 512 were early preterm. For the present study, we included all full-term and MLP children within this cohort whose smiling- and/or walking-age had been recorded by PCH professionals (*N* = 1241). All parents provided written informed consent. The study was approved by the Ethics Review Board of the University Medical Center Groningen (METc 2005/130). All procedures performed in the context of this study were in accordance with ethical standards of our institution and with the 1964 Helsinki Declaration.

### Developmental milestones: ‘smiling-age’ and ‘walking-age’

In the Netherlands, development of all children is monitored routinely by PCH physicians during scheduled well-child visits. During these visits, developmental milestones are assessed including “smiling back in response” and “walking independently”. For these two milestones, exact attainment ages are recorded, whereas for other milestones, it is only assessed if they have been attained or not. PCH physicians record the chronological attainment ages of these two milestones very accurately (in weeks and months, respectively) based upon parental report. Parents are not asked beforehand to determine attainment of these two milestones. Around the approximate attainment age, PCH physicians ask whether the milestone is attained and at what age. In general, for full-term children, for smiling this approximate attainment age is 6 weeks and for walking 18 months. We calculated the corrected smiling-age and walking-age by subtracting the number of weeks a child was born too early from the chronological age. We did so for all MLP children.

### Ages and stages questionnaire

Development at age 4 (range 43–49 months calendar age) was assessed by parents using the Dutch version of the Ages and Stages Questionnaire (ASQ) 48 months’ form. The ASQ was completed after informed consent was provided, as part of the LOLLIPOP study; the ASQ is not part of routine care in PCHCs in the Netherlands. The Dutch ASQ is recognized as a valid, reliable, cost-effective, fast and easy way to screen children for developmental delay, with high sensitivity (89%) and acceptable specificity (80%) for the detection of school problems [19]. The ASQ 48 months’ form contains 30 items in 5 developmental domains: communication, gross motor, fine motor, problem solving and personal-social functioning [19]. All five domains add up to a total ASQ score which indicates a child’s general development. A score below − 2 standard deviations (SDS) for the reference group of Dutch full-term children was considered to be below the threshold, both for the ASQ total score and the separate domains.

### Gestational age and covariates

GA was extracted from medical files and verified by early ultrasound measurements during pregnancy in over 95% of all cases. Children whose GA could not be reliably established were excluded. Other covariates were gender, ethnicity (defined as Dutch versus non-Dutch), being born small-for-gestational age (SGA; defined as <P10 on Dutch Kloosterman growth curves), [20] and maternal educational level (defined as low [≤12 years] versus middle/high [> 12 years]); these factors have been shown to be related to developmental outcomes [21–23]. Information on all covariates was extracted from a general questionnaire and matched to both hospital files and PCHCs files.

### Statistical analyses

First, we compared background characteristics of MLP and full-term children, using chi- square tests for binary variables and Mann-Whitney-U tests for continuous variables. Next, we compared the median attainment ages of smiling and walking between the two GA categories using Mann-Whitney-U tests. Third, we performed univariable logistic regression analyses, with corrected smiling-age and walking-age as determinants and the dichotomized score on ASQ domains as outcomes. The children who reached the milestones first (after correction for GA in the MLP group) were defined as the reference, to identify the added risk that each week later attainment age would give. We stratified these analyses by GA category. In the final step we performed multivariable logistic regression analyses and included gender, ethnicity, SGA and maternal educational level for found statistically significant associations in the univariable regression analyses. *P*-values below 0.05 were considered statistically significant. Analyses were conducted with SPSS version 26.0 (IBM Corp., Armonk, NY, USA).

## Results

Participant characteristics are presented in Table 1. Non-response in the developmental assessments differed for a few of the participant characteristics. Children without a recorded smiling-age more often had a non-Dutch mother (13.6% vs. 8.8%; *p* < 0.001). Children without a recorded walking-age more often had a lower educated mother (61.4% vs. 66.8%; *p* = 0.025).

**Table 1:** Characteristics of children with a recorded smiling-age^a^ and/or walking-age^a^ (*N* = 1241)

	Moderately-late preterm (***N*** = 769)	Full-term (***N*** = 472)	***P***-value
Gestational age (wks); mean (SD)	34.0 (1.04)	39.6 (1.00)	**< 0.001**
Birth weight (g); mean (SD)	2240 (469)	3543 (493)	**< 0.001**
SGA, N (%)	65 (8.4)	32 (6.8)	0.25
Male gender, N (%)	448 (58.3)	222 (47.0)	**< 0.001**
Ethnicity (*N* = 1420)			0.80
Dutch, N (%)	630 (94.7)	387 (95.1)	
Non Dutch, N (%)	35 (5.3)	20 (4.9)	
Maternal educational level (*N* = 1438)			0.20
High, N (%)	71 (25.5)	120 (29.1)	
Low/middle, N (%)	500 (74.5)	293 (70.9)	
Recorded smiling-age, N (%)	313 (40.7)	201 (42.6)	0.51
Recorded walking-age, N (%)	685 (89.1)	425 (90.0)	0.59
Both smiling-age and walking-age recorded, N (%)	229 (29.8)	154 (32.6)	0.29
ASQ at age 4 below the threshold, N (%)			
Communication	61 (7.9)	24 (5.1)	0.058
Personal-Social	34 (4.4)	9 (1.9)	**0.018**
Problem Solving	30 (3.9)	13 (2.8)	0.29
Fine Motor	46 (6.0)	11 (2.3)	**0.003**
Gross Motor	35 (4.6)	19 (4.0)	0.64
Total score	47 (6.1)	15 (3.2)	**0.021**

*SGA* Small-for-gestational-age, <P10 on Dutch Kloosterman Curves. Maternal educational level: Low < 12 years formal education, Middle < 16 years, High 16+ years. *ASQ* Ages and Stages Questionnaire. ^a^Data for preterm children are corrected for gestational age. Bold printed *p*-values: < 0.05

### Associations of smiling-age and walking-age with developmental delay

**Fig. 1:**
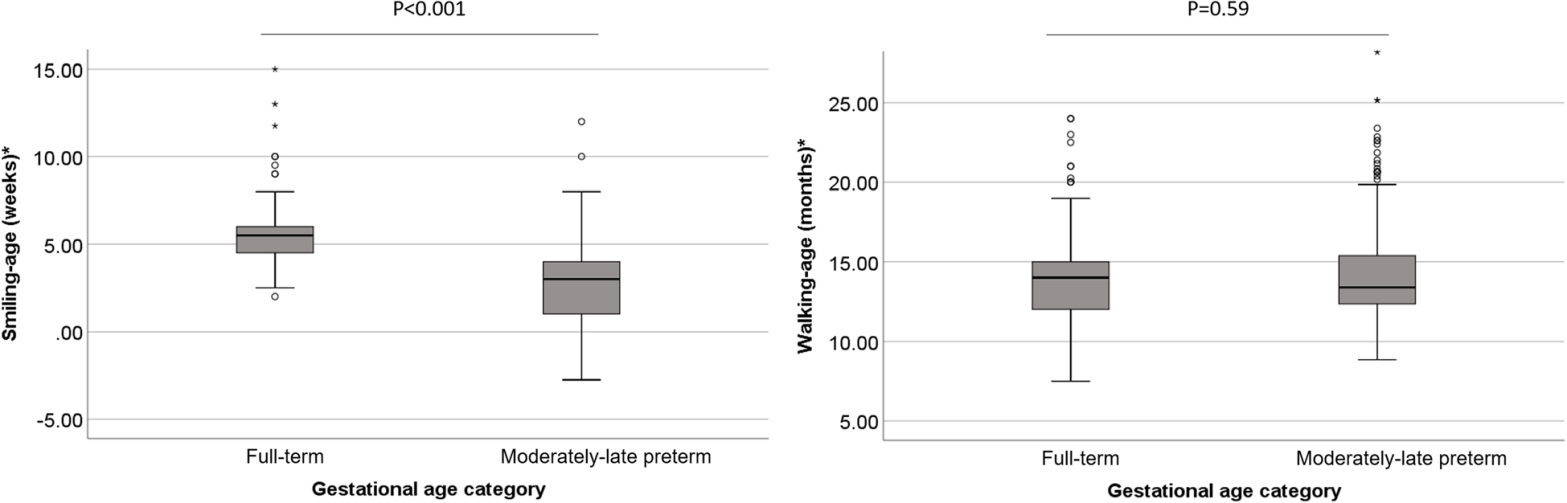
Median (range) smiling-age^ (left) and walking-age^ (right) for full-term and MLP children. In these box whisker plots, the boxes represent the interquartile ranges, the whiskers represent the range of all ages and the dots and stars represent outliers. ^ ages are corrected for gestational age in MLP children

In Fig. 1 we present the distribution of smiling-age and walking-age for the two GA categories. Median (range) smiling-age was 5.5 (2.0;15.0) weeks for full-term children and 3.0 (− 2.8;12.0) weeks for MLP children (*p* < 0.001). For walking age, median (range) for full-term children was 14.0 (7.5;24.0) months and for MLP children 13.4 (8.9;29.0) months (*p* = 0.59). Using corrected ages for MLP children thus resulted in ages that were within the normal ranges for full-term children. In Table 2 we provide the likelihood of ASQ domain and total scores below the threshold for each week later corrected smiling-age and each month later corrected walking-age, based on odds ratios (ORs). For smiling-age in MLP children, each week later smiling-age was associated with a 31, 43, 36 and 35% relative increased likelihood of personal-social, problem solving, gross motor problems, and general developmental delay, respectively. For full-term children no significant relationships were found. Combining the two GA categories in one model also did not result in statistically significant relationships.

**Table 2:** Likelihood of Ages and Stages Questionnaire domain and total scores below the threshold (<−2 SD) at age 4 per week/month later age at which a child first smiles (in weeks)^a^ and walks (in months)^a^: odds ratios (OR) crude and stratified by gestational age group

**Smiling-age (weeks)**									
	**All children** *N* = 514			**Full-term only (GA 38–42)** *N* = 201			**Moderately-late preterm only (GA 32–36)**^a^ *N* = 313		
**ASQ**	**OR**	**95%-CI**	***P*-value**	**OR**	**95%-CI**	***P*-value**	**OR**	**95%-CI**	***P*-value**
Communication	1.10	0.97–1.24	0.13	1.07	0.74–1.54	0.71	1.22	0.98–1.52	0.080
Personal-Social	1.04	0.90–1.20	0.64	1.02	0.62–1.66	0.95	**1.31**	**1.02–1.67**	**0.031**
Problem Solving	1.09	0.93–1.28	0.29	1.08	0.68–1.70	0.75	**1.43**	**1.05–1.95**	**0.022**
Fine Motor	0.93	0.82–1.07	0.31	1.28	0.72–2.25	0.40	1.16	0.93–1.46	0.18
Gross Motor	1.05	0.92–1.19	0.47	1.15	0.80–1.66	0.46	**1.36**	**1.03–1.80**	**0.031**
Total Score	1.05	0.93–1.19	0.42	1.24	0.88–1.76	0.23	**1.35**	**1.08–1.67**	**0.007**
**Walking-age (months)**									
	**All children** *N* = 1110			**Full-term only (GA 38–42)** *N* = 425			**Moderately-late preterm only (GA 32–36)**^a^ *N* = 685		
**ASQ**	**OR**	**95%-CI**	***P*-value**	**OR**	**95%-CI**	***P*-value**	**OR**	**95%-CI**	***P*-value**
Communication	**1.06**	**0.01–1.13**	**0.029**	1.14	0.97–1.34	0.12	1.06	0.99–1.13	0.12
Personal-Social	**1.12**	**1.05–1.20**	**0.001**	**1.33**	**1.07–1.65**	**0.010**	**1.10**	**1.01–1.18**	**0.021**
Problem Solving	1.06	0.99–1.14	0.099	0.93	0.71–1.23	0.63	1.06	0.97–1.15	0.18
Fine Motor	**1.13**	**1.06–1.20**	**< 0.001**	1.20	0.98–1.48	0.083	1.07	1.00–1.15	0.066
Gross Motor	**1.21**	**1.13–1.30**	**< 0.001**	**1.35**	**1.14–1.60**	**< 0.001**	**1.15**	**1.06–1.25**	**0.001**
Total Score	**1.16**	**1.09–1.23**	**< 0.001**	*1.26*	*1.04–1.52*	*0.016*	**1.13**	**1.05–1.22**	**0.002**

*ASQ* Ages and Stages Questionnaire, *OR* Odds Ratio, *95%-CI* 95% Confidence Interval, *GA* Gestational age. ^a^Data for preterm children are corrected for gestational age. Bold printed indicate that results remained similar after adjustment for gender, small-for-gestational age status, maternal educational level and ethnicity. Italic printed indicate that results lost statistical significance after adjustment for these covariates

Regarding walking-age of MLP children, each month later walking-age was associated with a 10, 15 and 13% relative increased likelihood of personal-social and gross motor problems, as well as a general developmental delay, respectively. For full-term children, we also identified increased likelihoods of delays in these domains. Combining the two GA categories in one model resulted in additional increased likelihoods in the communication and fine motor domains. All results remained similar after adjustment for gender, SGA status, maternal educational level and ethnicity, except for the association between walking-age and general developmental delay in full-term children.

## Discussion

This study demonstrated that in MLP children each week later corrected smiling-age increased the likelihood of personal social, problem solving, gross motor problems, and general developmental delay at school entry. Similarly, each month later corrected walking age increased the likelihood of personal-social and gross motor problems, and general developmental delay at school entry. Corrected smiling-ages and walking-ages were within the normal ranges of full-term children. For full-term children, we found no associations of smiling-age with developmental delay at school entry, but we did identify increased likelihoods of later walking-age with later personal-social and gross motor domains, and general developmental delay. In our multivariable models most increased likelihoods of developmental delay remained significant after adjustment for other covariates.

We found different associations for smiling-age and walking-age with distinct developmental domains at school entry. For a later attainment of smiling we found an increased likelihood of developmental delay for MLP children only. The only further available study by Flensborg-Madsen et al. on smiling reports that for full-term children smiling-age is predictive of IQ at 34 years of age [24]. This contrasts with our results, as we did not find any associations for full-term children, which may be due to our considerably smaller study sample. For MLP children, we did not find any studies reporting on the relationship between smiling-age and developmental delay later on. Nonetheless, the associations we identified with the distinct ASQ domains seem plausible, as relevant abilities such as communication, of which smiling-age may be an outing, are generally believed to underlie or precede several developmental skills, including cognition [25].

For a later attainment of walking we found increased likelihoods of general developmental delay, and of delays in gross motor and personal-social functioning, for both full-term and MLP children. Other studies, regarding full-term children, also reported associations of walking-age with IQ and educational level in adolescence and adulthood [7, 24]. For MLP children, these associations were not reported before. Murray et al. provide an explanation for differences in associations of different types of milestones with multiple domains [8]. They speculate that varying stages of development involve different neuronal pathways, and the maturation of these pathways may affect brain regions associated with development in later life. The identified associations were stronger for full-term children than for MLP children. We speculate that whereas a delay in walking-age in full-term children may predict a more significant motor delay in later life, for MLP children a delay in walking-age is less predictive because cephalocaudal myelination occurs more slowly due to their immature central nervous system at birth. However, recognition of early markers of developmental delay could provide opportunities and targets for early interventions to enhance maturation of neuronal pathways and promote normal patterns of development.

We identified several associations between smiling-age, walking-age and several ASQ domains at school entry in MLP children, which may also relate to school readiness of these children. Internationally, the concept of school readiness is often defined as cognitive and behavioral outcomes at school entry [26] and encompasses emotional regulation and attention; areas where MLP children often have delays [27]. Although the ASQ does not include emotional regulation and attention, problem-solving skills and personal-social behavior are related to similar developmental skills [26, 27]. As such, smiling-age and walking-age may be valuable indicators of potential difficulties in school readiness in MLP children. If MLP children encounter a delay in attainment of these milestones, offering interventions, especially in a preschool environment, could stimulate skills needed for adequate school performance [26].

We found strong associations of outcomes for two milestones with developmental delay at school entry, especially for MLP children, which may indicate the need for more extensive early life monitoring of development. There are several other early life assessments of neurodevelopmental risk in preterm infants, for example the General Movements Assessment and developmental tests such as the Bayley Scales of Infant and Toddler Development. However, in clinical practice, in many countries including the Netherlands, it is not feasible to monitor every (preterm-born) child with such assessments, because of time constraints or requirements of specific expertise. For early preterm children many follow-up programs exist, including extensive neurodevelopmental testing, but for MLP children, such programs are often not cost-effective. Routine PCH assessments based on developmental milestones such as smiling-age and walking-age, take less time and are more feasible in a population-based setting. Potentially, these two milestones could be used as a first step in developmental assessment, with unfavorable outcomes serving as red flags for MLP children requiring a more intense developmental monitoring. This evidently requires further study.

### Strengths and limitations

Major strengths of our study were that we examined the role of developmental milestones early in life as potential red flags of an increased risk of developmental delay and potential identification of targets for preventive intervention, thereby enhancing chances for closer monitoring, earlier identification and early interventions. Second, we used data from a large community-based cohort, in which PCH data on development were included that had been recorded prospectively from birth onward at regularly scheduled consultations in PCHCs. Because of the community-based character of the LOLLIPOP study, we did not only include the MLP children admitted to a neonatal intensive care unit, but also the relatively “healthy” MLP children. This study creates awareness for developmental risks in MLP children, who do not routinely participate in follow-up programs. We add to the existing evidence by focusing on two important and easily obtainable developmental milestones based on parental report of attainment ages. This differs from using only physician observed attainment, which focuses on whether a milestone has been obtained or not, rather than on the timing of it. Our study shows that parental report of these milestones is associated with developmental delay at school entry. In addition, this is one of the first studies to do so for MLP children, which concerns a rather large group of children. We believe that our findings confirm the feasibility of follow-up with risk assessment using developmental milestones based on parental report in community-based settings.

We also recognize the limitations of our study. First, we used parent-reported information both for our determinants and our outcome measure, which may be suboptimal. However, it has been shown that parents know their own children very well and are known to accurately report on developmental milestone attainment [28]. In addition, the ASQ has proven to be a reliable screening tool to identify children at high risk of developmental delay [19] and studies have reported its good predictive validity for school difficulties and cognition at early school age [29]. Second, in 16% of all cases, smiling-age and/or walking-age was not recorded, with smiling-age recorded in a smaller part. Mothers of children with missing attainment ages were more likely to be of non-Dutch ethnicity and to have a lower educational level. The missing data could have been due to poor parental recall of their child’s attainment age or time constraints during PCH consultations. These missing data may have led to some less precise estimates of the studied associations. Moreover, it may have resulted in some underestimation of its strength due to the somewhat higher risks in these groups. Third, this study may have less power to detect associations in the full-term group, due to its relatively smaller size, its lower prevalence of developmental delay and smaller range of gestational ages. Finally, we conducted multiple comparisons, which may have led to chance findings, albeit *p*-values were mostly very small and were also significant with Bonferroni correction.

### Implications

A potential next step for research could include studying whether MLP children with delays on these two developmental milestones (i.e. smiling-age after 8 weeks and/or walking-age after 18 months corrected age) actually experience a developmental delay at school-age. Moreover, studies should focus on associations between developmental milestones and in-depth developmental testing, to identify which domains are most affected. In addition, a more sophisticated prediction model could be developed, for example a dynamic prediction model including these and other milestones, and other relevant characteristics (such as socio-economic status or Apgar score) that are collected at birth and during the life course. Such a model could preferably be used at various ages to estimate risks of developmental delay, repeatedly, enhancing to adjust medical decision making during the life course of these high-risk children.

For clinical practice, MLP children with delays on these two developmental milestones may benefit from more extensive follow-up and early interventions. Studies have reported that early intervention indeed improves neurodevelopmental outcomes [30]. Thus, when a child has a late smiling-age, a first step could be to offer additional follow-up appointments and provide parents with tips and tricks on how to stimulate interaction with their child. When a child has a late walking-age, potential interventions include physiotherapy or additional interaction with other children in a pre-school environment. Evaluation studies could provide more insight in the effectiveness of targeted interventions related to attainment ages of specific milestones in MLP children. In addition, community-based health professionals may pay more attention to recording smiling-age, because of its value as an important early developmental milestone in monitoring MLP children specifically.

## Conclusions

In conclusion, corrected smiling-age and walking-age of MLP children are mostly within normal ranges. However, within this MLP group later attainment of these two milestones still is associated with developmental delay on several domains upon school entry. Pediatricians and PCH physicians could use these two developmental milestones as red flags, indicating a need to monitor the children with late attainment ages more closely or offer them more thorough assessments or interventions.

## Data Availability

The datasets generated and/or analysed during the current study are not publicly available because the participant consent for the collection of data did not explicitly or implicitly include details of sharing their anonymized data. Due to the sensitivity of the data and the restrictions from the informed consent, the data will not be stored at a public repository, but are available from the corresponding author on reasonable request.

## Abbreviations

MLP: Moderately-late preterm
GA: Gestational Age
IQ: Intelligence Quotient
LOLLIPOP: Longitudinal Preterm Outcome Project
PCH: Preventive Child Healthcare
PCHCs: Preventive Child Healthcare Centers
ASQ: Ages and Stages Questionnaire
SDS: Standard Deviations
SGA: Small for Gestational Age
ORs: Odds Ratios
